# Electronic phase shift measurement for the determination of acoustic wave DOA using single MEMS biomimetic sensor

**DOI:** 10.1038/s41598-020-69563-1

**Published:** 2020-07-29

**Authors:** Renato C. Rabelo, Fabio D. Alves, Gamani Karunasiri

**Affiliations:** Department of Physics, Naval Postgraduate School, Monterey, CA 93943 USA

**Keywords:** Biological physics, Electronics, photonics and device physics

## Abstract

MEMS acoustic sensors have been developed to mimic the highly-accurate sound-locating system of the *Ormia ochracea* fly, which detects sound wavelengths much larger than its hearing organ. A typical *ormia*-based MEMS directional sound sensor possesses two coupled wings that vibrate in response to sound according to a superposition of its two main resonant modes, rocking and bending. Vibrations are transduced into electronic signals by interdigitated comb finger capacitors at each wing’s end along with a capacitance measuring circuitry. A sensor designed to exhibit resonant modes closely placed in frequency, enhancing their coupling, was operated with a closed cavity behind the wings. Simultaneous and independent measurements of electronic signals generated at each of the single sensor wings were used to determine incident sound direction of arrival (DOA). DOA was found proportional to the phase shift between them and to the difference over the sum of their amplitudes as well. Single sensor phase shift DOA measurement presented a resolution better than 3° for sound pressure levels of 25 mPa or greater. These results indicate that a single sensor operating in closed-cavity configuration can provide hemispherical unambiguous direction of arrival of sound waves which wavelength is much larger than the sensor size.

For many animal species, perceiving the direction under which a sensorial stimulus occurs is key for survival and reproduction. Considering auditory stimuli, cues used for these tasks rely on small differences in the pressure disturbances detected by physically separated sensory organs, either in amplitude, time of arrival or both. When the animal size is comparable to the sound wavelength in air, the required physical separation capable of making the differences perceivable by the nervous cells, processing or transmitting the detected stimulus, is naturally assured. For species of much smaller dimensions, the physical impediments of having sufficiently separated sound sensors pushed evolution towards using different mechanisms of detecting and processing the sound waves much needed for their basic survival tasks^1^. There are many insects that rely on phonotaxis in order to complete their reproductive cycles^2–5^. Usually the process involves parasitism of different species’ male hosts by the larvae deposited on or near them. Males give off their location by emitting mating calls, directed to the female individuals of their own species, but those are also heard by female individuals of the parasitic species. This happens in low light conditions, in order to avoid visually based predators, therefore, directional sound sensory capabilities are required for both species^3–7^.

There are a number of studies concerned with one species of fly, *Ormia ochracea*, which show the ability of finding the chirping host for their larvae with high accuracy, despite its sensory organ being much smaller than the wavelength^5–8^. Miles et al*.*^9,10^ found that the mechanical coupling between two tympana mediated by a semi-rigid cuticle was responsible for bringing those stimuli amplitude and time differences to within reasonable working ranges for the neuronal cells involved, enabling the parasitic fly to acoustically locate the host for its larvae. As a consequence of these findings, research and development of acoustic sensors based on micro-electro-mechanical systems (MEMS) mimicking the directional sound sensory capability of the fly’s hearing organ in a small form factor have been explored^11–23^.

A typical Ormia-based MEMS directional sound sensor consists of two wings, connected by a bridge in the middle and the entire structure is connected to a substrate using two torsional legs. The structure exhibits two vibration modes: rocking, in which wings oscillate out of phase and bending, presenting in phase oscillations. When an acoustic pressure wave is incident on the sensor, the response of the mechanical structure is a linear combination of its vibration modes^9^. On the female fly hearing organ, each of the tympanal membranes present exclusive bilaterally-symmetric mechanoreceptive structures, the auditory apodeme and the bulba acustica^8^. Those are responsible for the transduction of each tympanal membranes mechanical vibrations into neuronal electrical pulses. This way, mechanical displacements of each tympanal membrane resulting from acoustic coupling are simultaneously and independently encoded. Likewise, on the MEMS directional acoustic sensors a transduction mechanism must be employed to convert each of the wings’ vibrations into electronic signals.

Several transduction mechanisms were envisioned. The optoelectronic mechanism, based on the angular position of a monochromatic light beam diffracted off a grating ^13,17^, as well as the one based on fiber-optic low-coherence light interferometry^12,16,19^ both take advantage of the high-sensitivity, high-resolution provided by photonic techniques, however at the cost of a higher complexity optical mounting or signal processing. An alternative technique was explored using an integrated PZT film deposited on the sensor die^18,20^. This was found to be less sensitive than other techniques at the expense of increased complexity on the microfabrication process by adding the piezoelectric film deposition, polling, patterning and etching. In order to simplify the fabrication process our group opted to employ interdigitated comb finger capacitors integrated at the edges of the wings^14,15^. These are patterned and etched during the same processes of patterning and releasing the wings. A capacitance measuring circuit completes the full transduction from mechanical displacement to electronically measurable signal. The combs were designed using a fishbone architecture to increase structural rigidity while attaining appreciable capacitance change^22^. Similar transduction mechanism was also employed by other research groups^21^ in their devices.

Aiming at finding the direction of arrival of acoustic stimuli, previous research works employed either a MEMS directional sound sensor along with a calibrated microphone^21^ or by using two MEMS directional sound sensors^22^. The latter was operated at the bending mode under sound interaction on both sides of the wings, similar to that of a pressure gradient microphone, giving a cosine directivity pattern. In order to extract the acoustic wave direction of arrival, such arrangements were needed to eliminate the unknown sound pressure, in the first one, or the angular ambiguity, as in the second. In this paper, in close similarity to the fly’s hearing organ, the use of a single MEMS sensor for accurately determining the direction of sound is demonstrated.

## Results

### Sensor design and analysis

**Figure 1:**
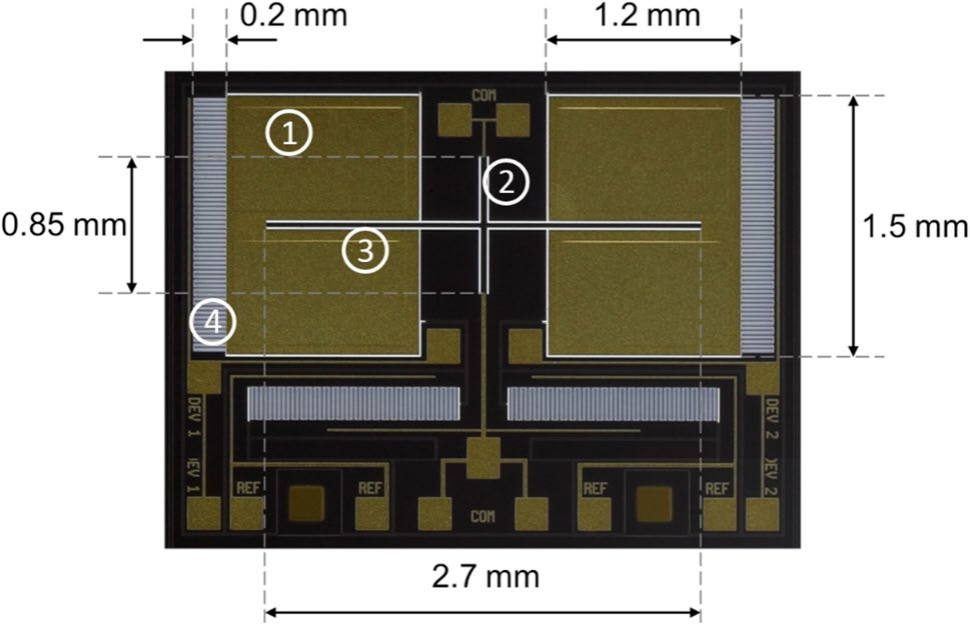
Optical micrograph of the tested MEMS sensor. ➀ Wing—main movable surface due to acoustic coupling. ➁ Leg—beam connecting the bridge and all freestanding membranes to the substrate; it also acts as a torsional spring. ➂ Bridge—beam connecting two opposite wings, mechanically coupling them. ➃ Interdigitated comb finger capacitors transduce wings’ mechanical displacements into capacitance variation.

The MEMS sensor employed in this paper consists of two 1.2 × 1.5 mm^2^ wings, coupled by a 2.7 mm long bridge. Two legs, perpendicular to the bridge, connect the entire mechanical structure to the substrate and also act as torsional springs. A micrograph of the device surface is shown in Fig. 1. An additional diagram is provided on Supplementary Fig. S1 in which it is possible to perceive the displacements associated with each resonant mode.

The fabrication used a 400 μm-thick silicon-on-insulator (SOI) wafer, in which a 25 μm-thick device layer sits on top of a 1 μm-thick oxide layer. The comb finger capacitors were fabricated at the edges of the wings and details of the comb structure can be found in Downey^23^. SEM images detailing the fingers are provided in the Supplementary Fig. S2. Fabrication was performed by a commercial foundry (MEMSCAP Inc.) through a multiuser single-wafer process (SOIMUMPS).

The mechanical response of the MEMS sensor to incident sound may be modeled similarly to the approach developed by Miles et al*.* for the fly’s hearing organ using a lumped parameter coupled system^9^. The motion of each wing under steady-state harmonic excitation can be treated as a linear combination of two natural modes of vibrations, the rocking mode and the bending mode. The rocking mode motion results from pressure differences on the two wing’s surfaces due to the oblique incidence of the sound waves. The two wings will suffer displacements in opposite directions twisting the two legs, ensuing a phase shift of π radians between them. Therefore, this mode behaves as if the bridge were rigid. The bending mode is excited by the pressure of the incident sound with wings moving in phase by bending the bridge connecting them. The actual motion of the wings resulting from acoustic excitation at a given angular frequency ω will be a linear combination of these two natural modes. The degree of coupling between the two modes depends on their proximity in frequency as well as their bandwidths. The resulting displacement for each of the two wings can be expressed as^9^

1 $$\begin{array}{*{20}c} {x_{1} \left( t \right) = A_{b} \sin \left( {\omega t + \phi_{b} } \right) + A_{r} \cos \left( {\omega t + \phi_{r} } \right)} \\ {x_{2} \left( t \right) = A_{b} \sin \left( {\omega t + \phi_{b} } \right) - A_{r} \cos \left( {\omega t + \phi_{r} } \right),} \\ \end{array}$$

where $x_{i} \left( {i = 1,2} \right)$ are the ipsilateral and contralateral wing displacements, $A_{j}$ and $\phi_{j}$ are the normal mode amplitudes and phases, respectively ($j = b,r$). The subscripts $b$ and $r$ stand for bending and rocking modes, respectively. The Eq. (1) shows that two modes add constructively on one side while destructively on the other side giving two different oscillation amplitudes. This behavior is due to in phase and out of phase motions of bending and rocking modes of the MEMS structure.

By solving coupled differential equations of motion as detailed in Miles et al*.*^5^, taking into account the sound wavelength is much longer than wings’ separation, the amplitudes and phases of normal modes can be approximated as

2 $$\begin{aligned} A_{r} & = \frac{P s }{m}\left( {\frac{1}{{\sqrt {(\omega_{r}^{2} - \omega^{2} )^{2} + \left( {\gamma_{r} \omega } \right)^{2} } }}} \right)\frac{\omega d}{{2c}}\sin \theta , \\ A_{b} & = \frac{P s }{m}\left( {\frac{1}{{\sqrt {(\omega_{b}^{2} - \omega^{2} )^{2} + \left( {\gamma_{b} \omega } \right)^{2} } }}} \right), \\ \end{aligned}$$

and

3 $$\begin{aligned} \phi_{r} & = - atan\left( {\frac{{\gamma_{r} \omega }}{{\omega_{r}^{2} - \omega^{2} }}} \right), \\ \phi_{b} & = - atan\left( {\frac{{\gamma_{b} \omega }}{{\omega_{b}^{2} - \omega^{2} }}} \right), \\ \end{aligned}$$

where $\omega_{j}$ and $\gamma_{j}$ are the resonant angular frequencies and damping coefficients associated with of each of the normal modes ($j = b,r$), $\omega$ is the acoustic excitation angular frequency, $P$ is the acoustic wave pressure amplitude$, s$ is the wing surface area, $d$ is the separation of the two membranes, $\theta$ is the acoustic wave angle of incidence and $c$, the speed of sound.

It can be seen in Eq. (2) that both modal amplitudes depend on the sound pressure $P$ while only the rocking amplitude depends on the angle of incidence. Thus, the angle of incidence of sound may only be determined if there is prior knowledge or simultaneous measurement of the sound pressure level (SPL). One approach to overcome this requirement is by eliminating the dependence on $P$ by taking the ratio of the modal amplitudes from Eq. (2) as

4 $$\frac{{A_{r} }}{{A_{b} }} = \left( {\frac{{(\omega_{b}^{2} - \omega^{2} )^{2} + \left( {\gamma_{b} \omega } \right)^{2} }}{{(\omega_{r}^{2} - \omega^{2} )^{2} + \left( {\gamma_{r} \omega } \right)^{2} }}} \right)^{{{\raise0.7ex\hbox{$1$} \!\mathord{\left/ {\vphantom {1 2}}\right.\kern-\nulldelimiterspace} \!\lower0.7ex\hbox{$2$}}}} \frac{\omega d}{{2c}}\sin \theta .$$

Even though the modal amplitudes $A_{r}$ and $A_{b}$ may not be directly measured, they are implicit in the wing displacement signals $x_{1} \left( t \right)$ and $x_{2} \left( t \right)$ given in Eq. (1). Constructing two auxiliary signals from the individual wing displacements of Eq. (1), by adding and subtracting them as

5 $$\begin{array}{*{20}c} {x_{1} \left( t \right) + x_{2} \left( t \right) = 2A_{b} \sin \left( {\omega t + \phi_{b} } \right),} \\ {x_{1} \left( t \right) - x_{2} \left( t \right) = 2A_{r} \cos \left( {\omega t + \phi_{r} } \right).} \\ \end{array}$$

The auxiliary signal amplitudes are multiples of $A_{r}$ and $A_{b}$ and their ratio may be readily calculated to extract the direction of arrival (DOA) of the incident sound wave. Not only the ratio of difference over sum carries information about the angle of incidence of the acoustic stimulus but also the phase shift between the signals $x_{1} \left( t \right)$ and $x_{2} \left( t \right)$. Since the two signals represented in each of the lines in Eq. (1) are linear combinations of harmonic signals, $x_{1} \left( t \right)$ and $x_{2} \left( t \right)$ are themselves also harmonic and can be represented as

6 $$\begin{array}{*{20}c} {x_{1} \left( t \right) = A_{1} \cos \left( {\omega t + \phi_{1} } \right),} \\ {x_{2} \left( t \right) = A_{2} \cos \left( {\omega t + \phi_{2} } \right),} \\ \end{array}$$

with their amplitudes $A_{j}$ and phases $\phi_{j}$ ($j = 1,2$) being functions of the normal modes amplitudes and phases. It can be shown that phase shift between $x_{1} \left( t \right)$ and $x_{2} \left( t \right)$ in Eq. (6) can be expressed as

7 $${\Delta }\phi = \phi_{1} - \phi_{2} = atan\left( {\frac{{2\frac{{A_{r} }}{{A_{b} }}{\cos}\left( { \phi_{b} - \phi_{r} } \right)}}{{1 - \left( {\frac{{A_{r} }}{{A_{b} }}} \right)^{2} }}} \right),$$

and their amplitudes as

8 $$\begin{aligned} A_{1}^{2} & = A_{r}^{2} + A_{b}^{2} + 2A_{r} A_{b} \sin \left( {\phi_{b} - \phi_{r} } \right), \\ A_{2}^{2} & = A_{r}^{2} + A_{b}^{2} - 2A_{r} A_{b} \sin \left( {\phi_{b} - \phi_{r} } \right). \\ \end{aligned}$$

Equations (4) and (7) show that using either the ratio of modal vibration amplitudes or phase shift between wing displacements enables the determination of direction of arrival (DOA) of an acoustic wave using a single sensor.

### Single sensor assembly and characterization

**Figure 2:**
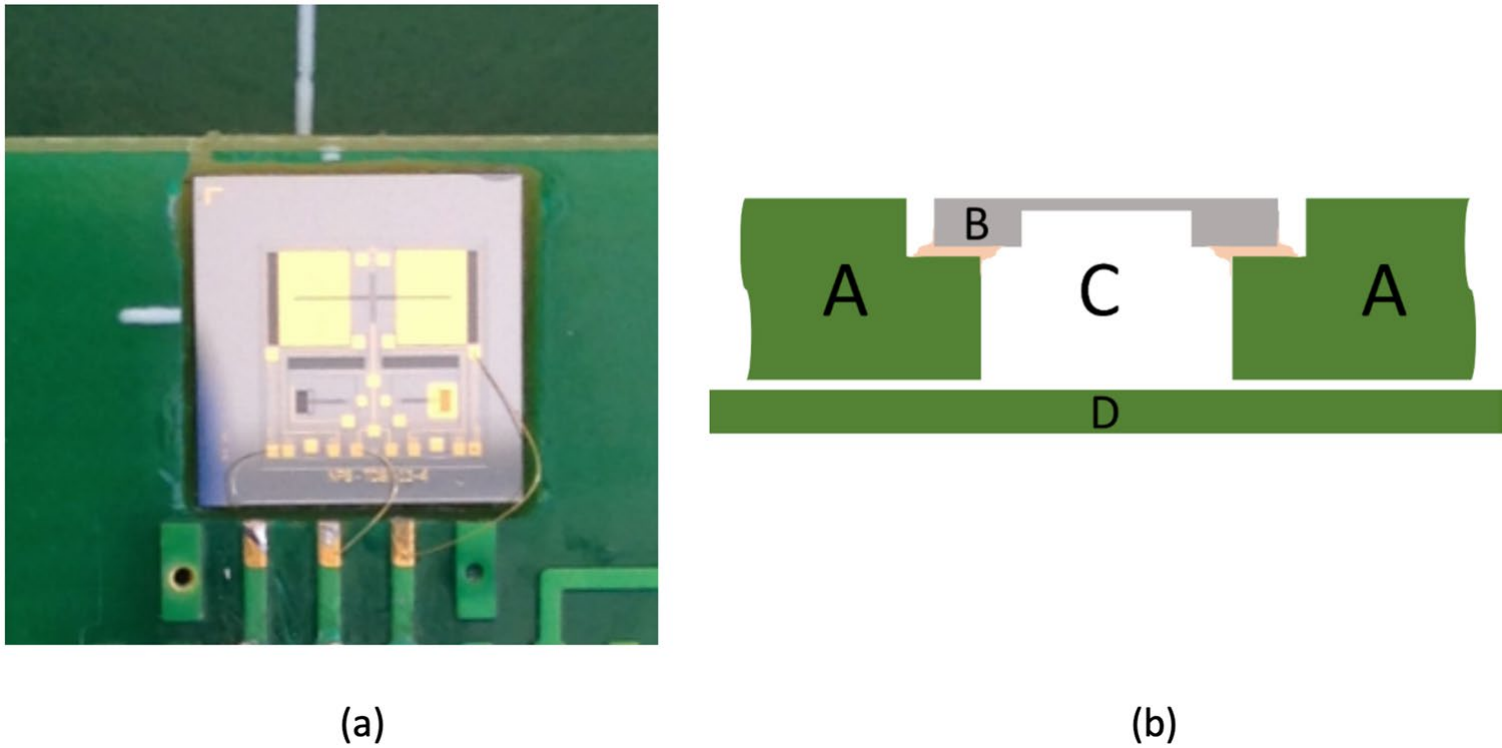
Sensor assembly on a pressed circuit board (PCB). Topview picture of the sensor die glued and wirebonded to the circuit board (**a**). Sideview schematic diagram of the sensor assembly (**b**): the sensor die (B) was glued on a top PCB board (A), over a through-hole (C); a separate PCB board piece (D) was attached to the bottom side configuring a back air cavity for the sensor. The space left between the two circuit boards (A and D) in the schematic is intended to convey the idea of imperfect sealing and physical link (air and pressure) between the cavity and the exterior medium.

The sensor die was glued on a 2.5 mm thick circuit board over a milled-through hole as schematically shown in Fig. 2. The through-hole was sealed using a piece of PCB attached to the opposite side of the circuit board creating an air cavity underneath the sensor. Since it was not glued, the cavity was not hermetically sealed. On previous works the through-hole was not covered, allowing the acoustic wave to interact on both sides (front and back) of the sensor wings, resulting a cosine directivity pattern similar to a pressure gradient microphone^14,15,22^.

**Figure 3:**
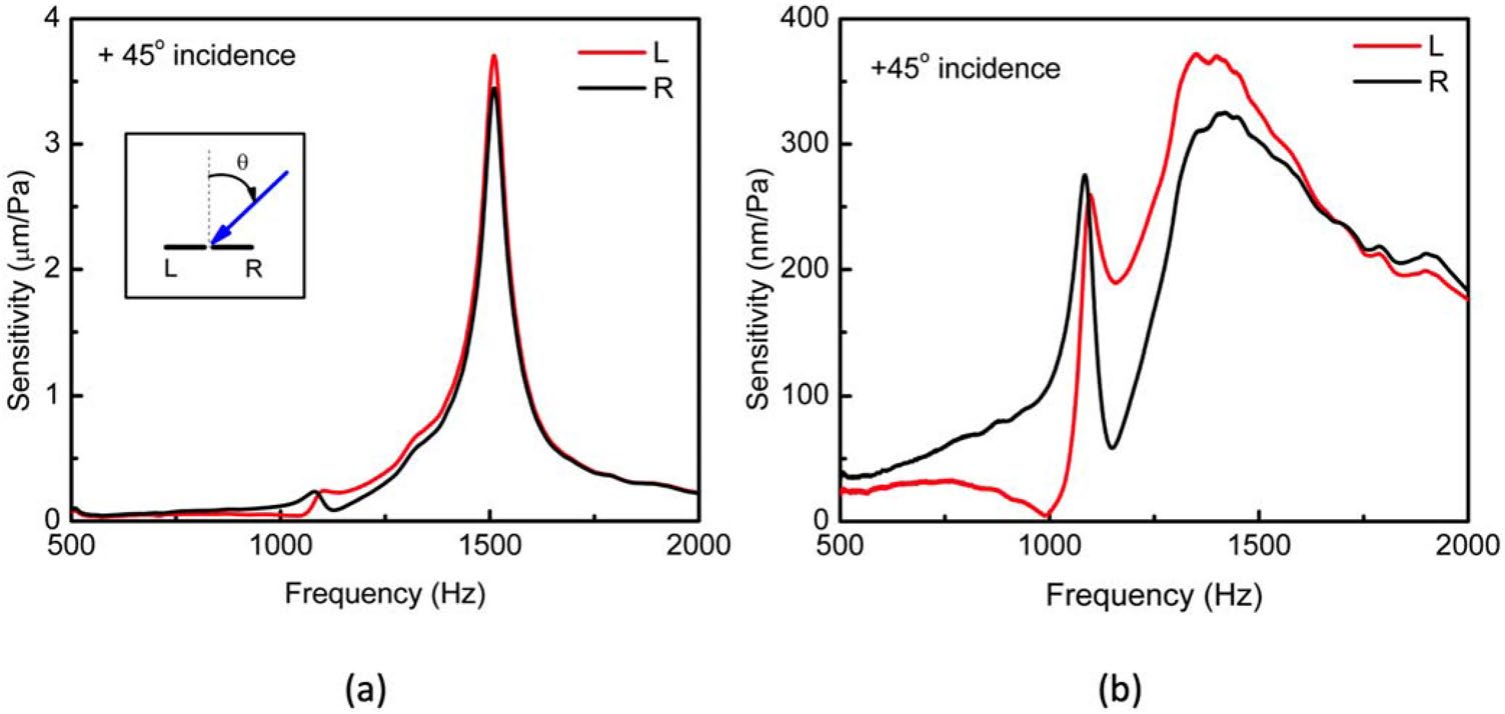
Sensor mechanical frequency response. Under excitation of a sound wave at oblique incidence with respect to the sensor, the frequency responses of the wings displacement per unit sound pressure level [${\text{m}} \cdot {\text{Pa}}^{ - 1}$] were measured (**a**) before closing the back cavity and (**b**) after attaching the backplate, closing the cavity. Inset in (**a**) displays the adopted reference system and nomenclature: angles are measured positive in the clockwise sense from the normal of the sensor. Left (L) and right (R) orientation are defined for an observer placed at the sensor, facing the sound source.

Figure 3 shows measured frequency responses of the sensor with open and closed back configurations using a Polytec OFV-5000 laser vibrometer installed inside an anechoic chamber when the angle of incidence of sound was about 45°. The open back configuration in Fig. 3a showed two resonance peaks at 1082 Hz (rocking mode) and 1510 Hz (bending mode). The peak widths (FWHM) were found to be about 50 Hz for both modes. When compared with the open back counterparts, the closed back configuration response presented in Fig. 3b, exhibit the peak at 1082 Hz with nearly the same height and width, while the one that was centered at 1510 Hz became broader and slightly downshifted. It can also be seen in Fig. 3b that a drastic reduction of bending mode amplitude occurs when the back is closed. The reduction of bending mode amplitude is most likely due to both wings pressing the air cavity simultaneously as opposed to being pressed by one wing at a time as with the rocking mode.

Thus, the rocking mode keeps the air volume in the cavity to be nearly the same while the bending mode compresses it. The increased spectral bandwidth observed on the bending mode response is associated with the losses induced by the viscous squeezed film damping as the two wings press the air volume in the cavity and force it through the gaps on the die trenches and between the two PCB boards. The relative proximity of bending and rocking mode response frequencies observed in Fig. 3a was intentional by design. The goal was to obtain adequate coupling between the modes, which in turn would result perceivably different wings’ oscillation amplitudes when under oblique sound incidence. If the coupling between the two modes was weak, the oscillation modes would behave almost independently, depending on which mode the excitation frequency was closer. Employing an excitation frequency closer to the bending mode would make the wings oscillate in phase with nearly the same amplitude, whereas excitation frequencies closer to the rocking mode would make them experience out of phase oscillations, also with nearly the same amplitude. In either case, it would be difficult to determine the direction of arrival (DOA) of sound utilizing a single sensor because the wings’ oscillation amplitudes or the phase shift between them remain almost the same. On the other hand, when the two modes are strongly coupled, wings’ oscillation amplitudes and phase shift at oblique sound incidence become perceivably distinct. This is due to the coupling term, expressed as the product of the two modal amplitudes in Eq. (8), resulting in different wing oscillation amplitudes.

**Figure 4:**
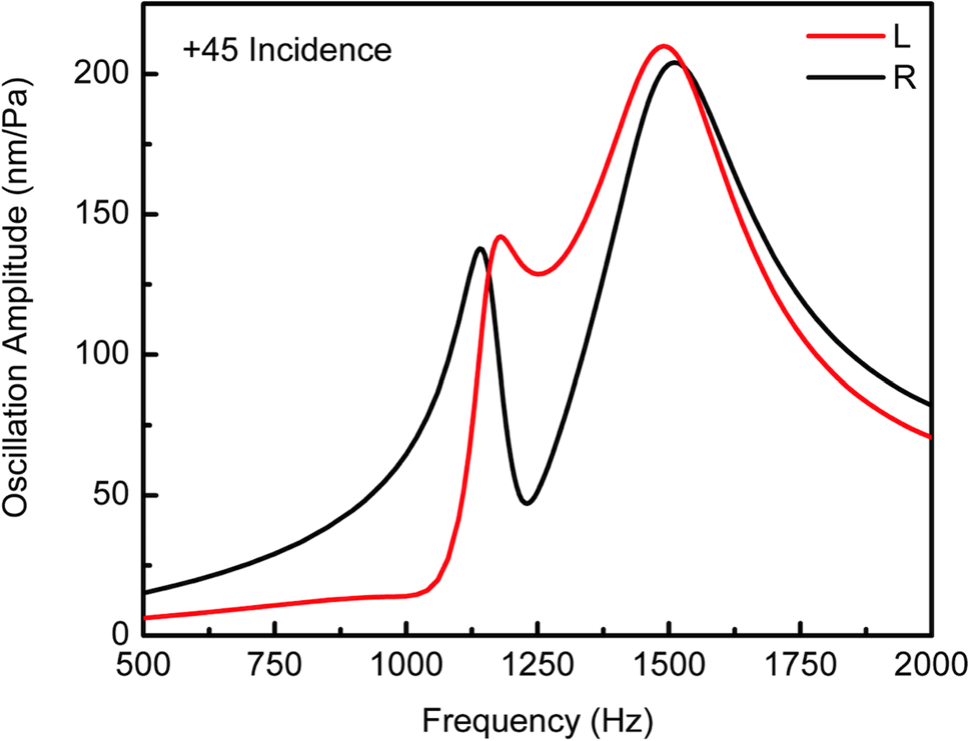
Simulated mechanical frequency response. Finite-element method simulation for sensor under similar acoustic excitation used in the vibrometer measurement (+ 45° oblique incidence). A small slit added to the cavity backplate modeled the air and pressure exchange of an imperfectly closed back cavity with the exterior medium. By controlling the slit size, it was possible to tune the bending mode contribution to the resulting wing oscillation amplitude, until it was deemed close enough to the measurement in Fig. 3b.

Observing Fig. 3a, obtained by sweeping the sound source frequency while keeping a constant SPL, it is possible to notice the peak oscillation amplitude at the rocking mode being one order of magnitude smaller than that of the bending mode. This difference is a direct consequence of the sound coupling from both sides of the wings^14^. In order to minimize the sound coupling from backside of the wings, a back plate was used. Closing the back of the sensor, increases the bending mode damping coefficient $\gamma_{b}$ and decreases the bending mode oscillation amplitude under the same SPL (see Eq. 2). The increase in $\gamma_{b}$ results in the bending mode spectral response broadening as well as downshift in the resonant peak position. No effect on the rocking mode oscillation amplitude as a result of increasing $\gamma_{b}$ is expected, as may also be seen by comparing experimentally obtained Fig. 3a, b. Closing the back of the cavity reduced the bending mode oscillation amplitude, broadening its spectral behavior and bringing the resonant modes’ responses a slightly closer, all favoring the use of a single sensor for determination of DOA. Simulations accounting for the sensor placement over the configured cavity were preformed to confirm the expected spectral trends and helped establish a framework to design future sensor systems. The simulated air cavity depth was set to 2 mm, closed by a 400 μm-thick hard plate on the back surface. Since the actual air cavity is not hermitically sealed, a small slit was used to simulate the air exchange between the obtained cavity and the exterior medium. Simulations were performed with finite-element method (FEM) using COMSOL Multiphysics acoustic, structural and thermoviscous physics modules. Figure 4 shows the simulated frequency response of the sensor system with backside closed for a + 45° degrees angle of incidence. It can be seen in Fig. 4 that the simulated frequency response agrees reasonably well with that of the measurement in Fig. 3b.

### Readout electronics

**Figure 5:**
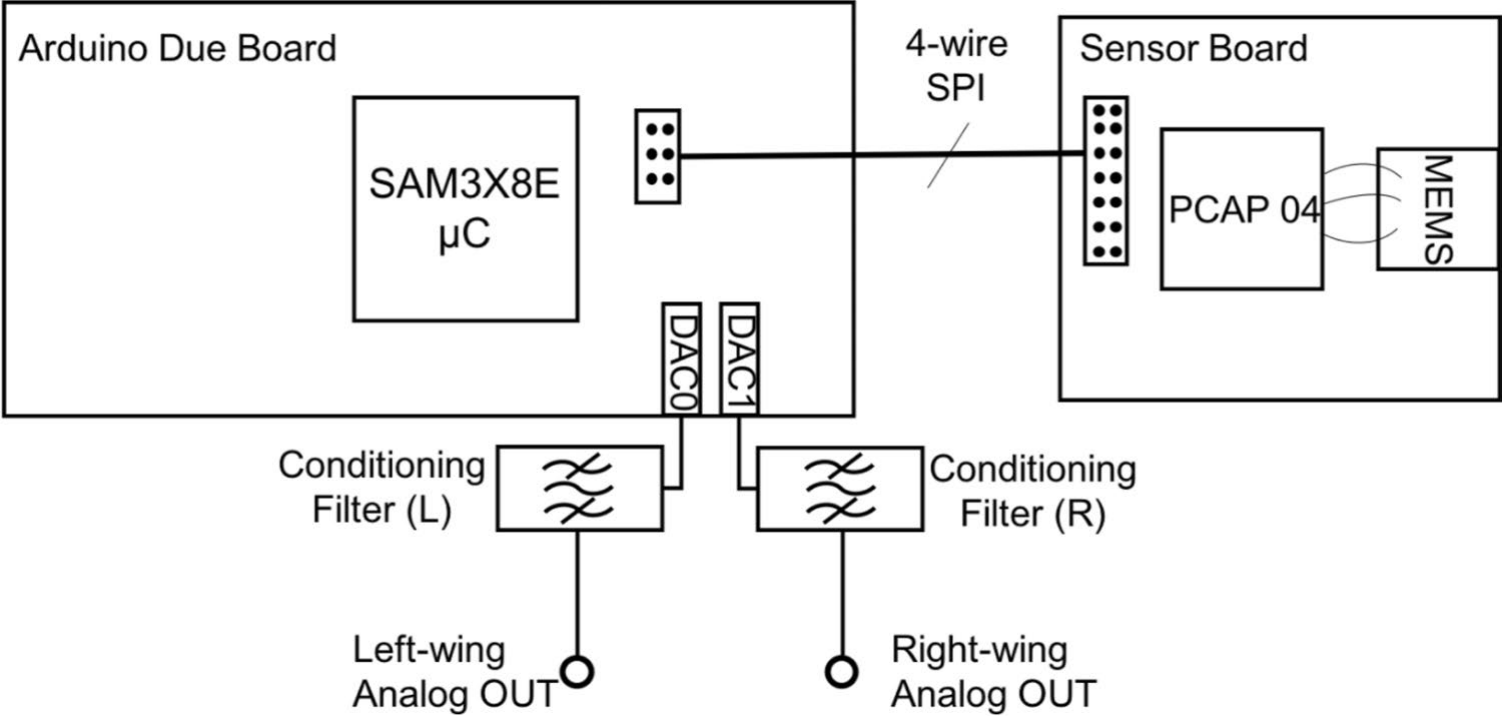
Electronic circuit diagram. The interdigitated comb finger capacitors associated with each wing were connected to one independent input of the PCAP04 capacitance-to-digital converter. Simultaneous wing capacitance readings were taken from PCAP04 output registers and transmitted through a four-wire serial peripheral interface (SPI) to a commercial 32-bit microcontroller evaluation board (Arduino Due). Each of the on-chip digital-to-analog converters (DAC) output a voltage associated with each wing mechanical displacement, resulting two analog signal channels: L (left) and R (right). Conditioning filter networks were used on each channel to further improve signal characteristics.

Pursuing the direction of arrival determination from an acoustic stimulus employing a single MEMS sensor requires the ability of simultaneously and independently detecting mechanical displacements associated with each of the wings. In order to do that, an integrated circuit, PCap04, from ACAM Messelektronik, now ams AG^24^ was employed. This IC is capable of simultaneously measuring either 6 single-ended or 3 differential capacitances generating a 32-bit digital output associated with each reading. The capacitance readings in the registers may be dynamically updated at up to a 50 kHz sample rate. The PCap04 was able to provide, with negligible crosstalk, the simultaneous readings of the capacitance values associated with each wing. Additional circuitry was needed to convert the two 32-bit digital words associated with each wing displacement to analog signals through a digital-to-analog conversion. This operation would have to be done fast enough to avoid loss of information or aliasing resulting from sampling the capacitance signal at a frequency smaller than the Nyquist lower limit. Since the frequencies of the sensor rocking and bending resonances were designed to be below 2000 Hz, the sampling rate of the IC is found to be adequate for reproducing the signal, producing roughly about 30 samples per period. The complete capacitance readout circuit assembled consisting of a 32-bit ARM Cortex SAM3X8E microcontroller (Arduino Due) integrated with PCAP04 is schematically shown in Fig. 5.

The microcontroller has two, on chip, 12-bit digital-to-analog converters (DAC) used to output as voltage signals the 32-bit capacitance readings performed by the PCAP04 integrated circuit. Communication between the microcontroller and the capacitance-to-digital integrated circuit was established through SPI connections available at both ends. The microcontroller on-chip DAC did not present high current fan out capacity and the addition of a buffering stage was needed. Moreover, the signal was still noticeably jigged (stepwise continuous) as a consequence of the DAC process and sampling frequency.

Therefore, an active DC block high-pass filter and an active 2nd order Butterworth low-pass filter as shown in Fig. 5 were added to each DAC output, which satisfied these remaining requirements. The high-pass cutoff frequency was about 160 Hz and the designed low-pass cutoff frequency was 3.3 kHz, placing the frequencies of interest in the resulting filter passband.

### Measurements

**Figure 6:**
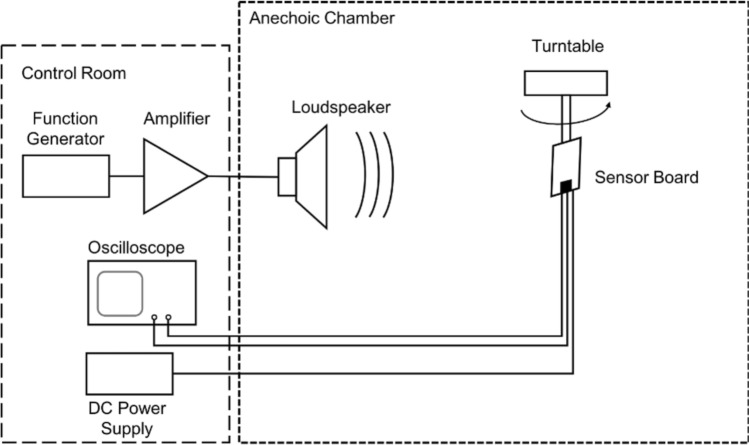
Frequency response and directionality setup diagram. Sensor board was attached to a turntable placed inside an anechoic chamber. A loudspeaker facing the turntable was utilized as the acoustic source. Sensor power supply, signal generating equipment and sensor signal reading instrumentation, represented here by an oscilloscope, were kept in the control room. For frequency response measurements, the signal was frequency-swept while keeping the sensor board fixed with respect to the source. For directionality, a single tone signal was applied while changing the angular position of the sensor with respect to the loudspeaker.

The sensor with the closed back along with the circuit board were mounted to a post and connected to a turntable inside an anechoic chamber, as schematically shown in Fig. 6, in order to measure its frequency response and its directionality.

**Figure 7:**
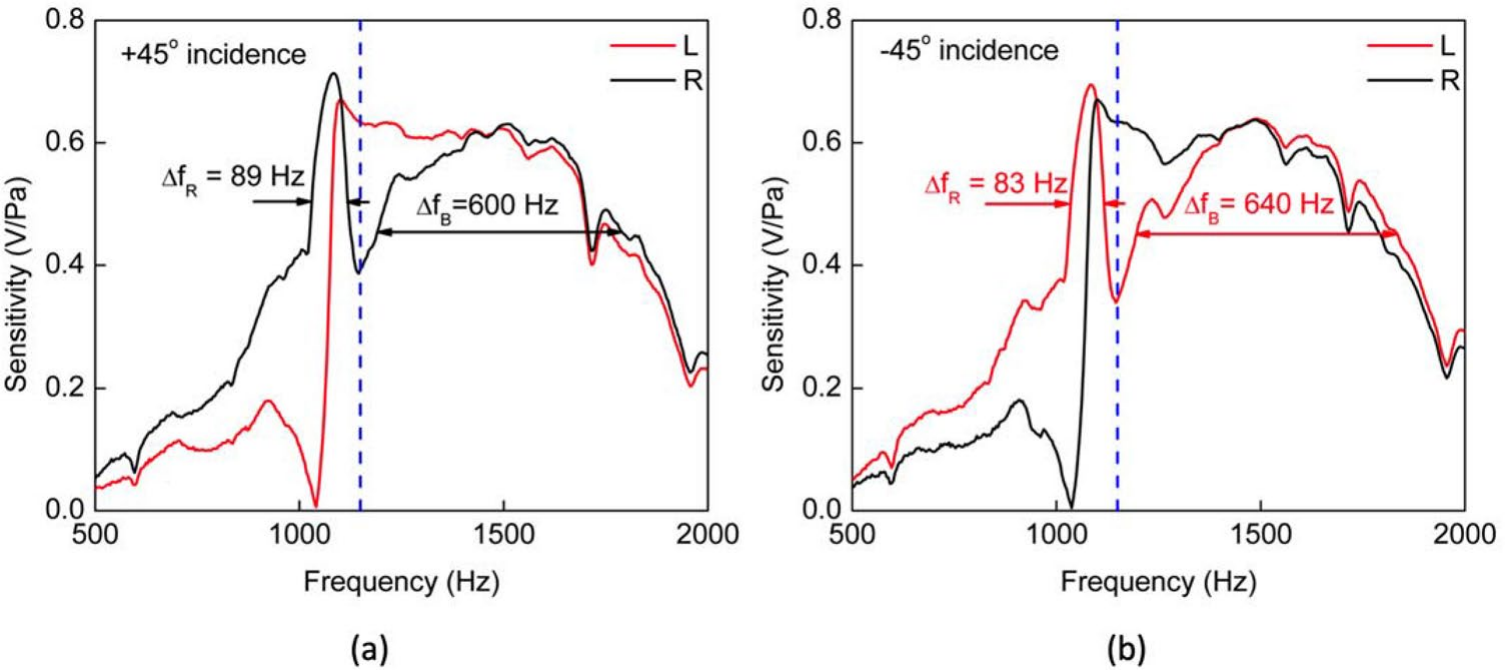
Electrically measured spectral responses. Electrical signal amplitudes obtained from each wing while the sensor was under swept-frequency acoustic excitation. Two angular orientations with respect to the incident wave were adopted: (**a**) + 45° and (**b**) − 45°. Reference system adopted is the same described on Fig. 3. Vertical blue dashed line locates the single tone frequency used in the directionality experiment (1150 Hz).

A fixed loudspeaker facing the turntable was used as the sound source which was excited using a function generator via an audio amplifier. The frequency response of the sensor was measured at two fixed angle positions (± 45°), by sweeping the frequency from 500 Hz up to 2000 Hz as shown in Fig. 7.

In accordance to what was expected, the bending mode sensitivity is not affected by the angular sense (positive or negative) of incident sound wave while the response at the rocking mode is strongly affected by which side the sound is incident upon. Also worth noting is the close resemblance of the spectral responses originated on the signal processing circuit (Fig. 7) with that measured with the laser vibrometer (Fig. 3b) and with that obtained from the FEM simulation (Fig. 4). In the directional behavior measurement, based either on the wings’ oscillation amplitudes or on the resulting phase shift as a function of incident angle, a single tone acoustic signal with frequency between the rocking mode and bending mode resonance peaks was chosen (1150 Hz). This frequency was chosen because it results in the greatest wings’ oscillation amplitude difference near the rocking mode.

**Figure 8:**
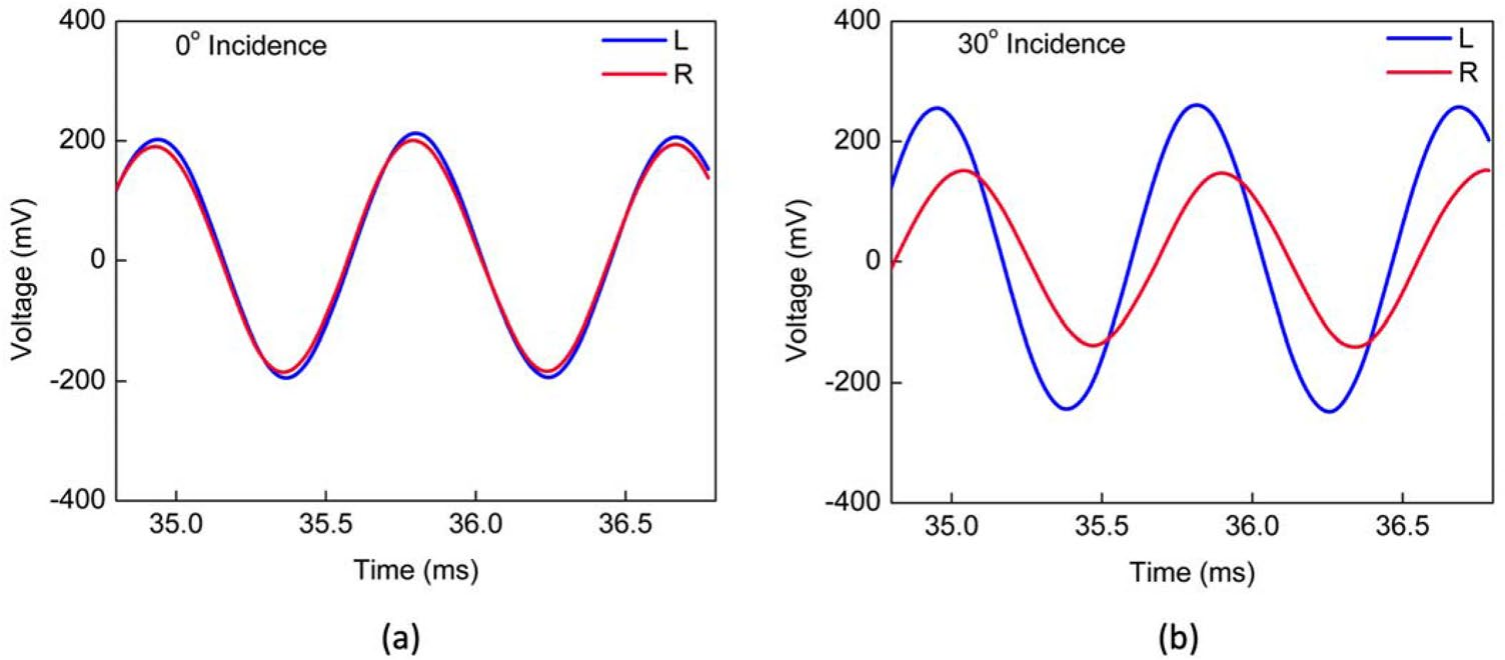
Recorded wings’ waveforms. Voltage signal associated with each wing, left (L, blue) and right (R, red), were stored each in one channel of an oscilloscope with the sensor under single tone (1150 Hz) sound excitation. The angle of incidence on the sensor was adjusted with the turntable for two distinct values: (**a**) normal incidence (0°); (**b**) incidence at 30°.

Figure 8a shows recorded waveforms from the two wings at normal incidence of sound. As expected, there is no phase or amplitude difference between the two signals since at normal incidence only the bending mode can be excited and the wings oscillate in phase. The waveforms on Fig. 8b were recorded for a 30°-incident sound wave. It clearly shows that the amplitudes and phases associated with the two wings are not the same due to coupling between the rocking and bending modes. For other angles of incidence, waveforms were recorded by rotating the turntable from − 90° to + 90° in 10° steps while keeping the same excitation amplitude (sound pressure level) and frequency. From the recorded waveforms, the phase shift between left and right signals was determined using their zero crossings. In order to compare direction of arrival (DOA) obtained from the phase shift with that of the amplitude ratio, the two signals, left (L) and right (R) were used to construct the auxiliary signals “sum” (L + R) and “difference” (L − R) for every turntable position.

**Figure 9:**
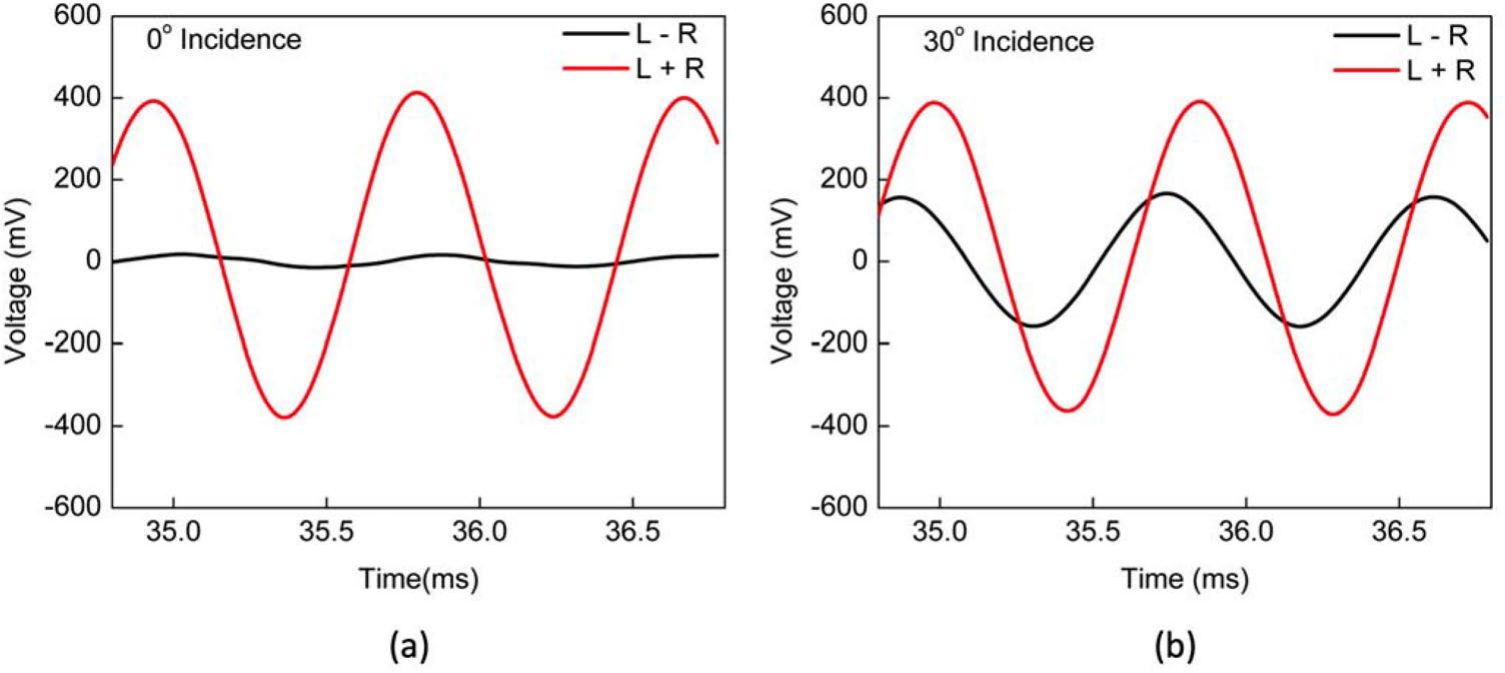
Auxiliary signals. Sum (L + R, red) and difference (L − R, black) auxiliary signals constructed from recorded wings’ waveforms. The procedure is exemplified for the two angular orientations shown in Fig. 8: (**a**) normal incidence (0°); (**b**) incidence at 30°. The obtained signal amplitudes are proportional to bending and rocking mode oscillation amplitudes and their ratio can recover the acoustic wave angle of incidence according to Eq. (4).

**Figure 10:**
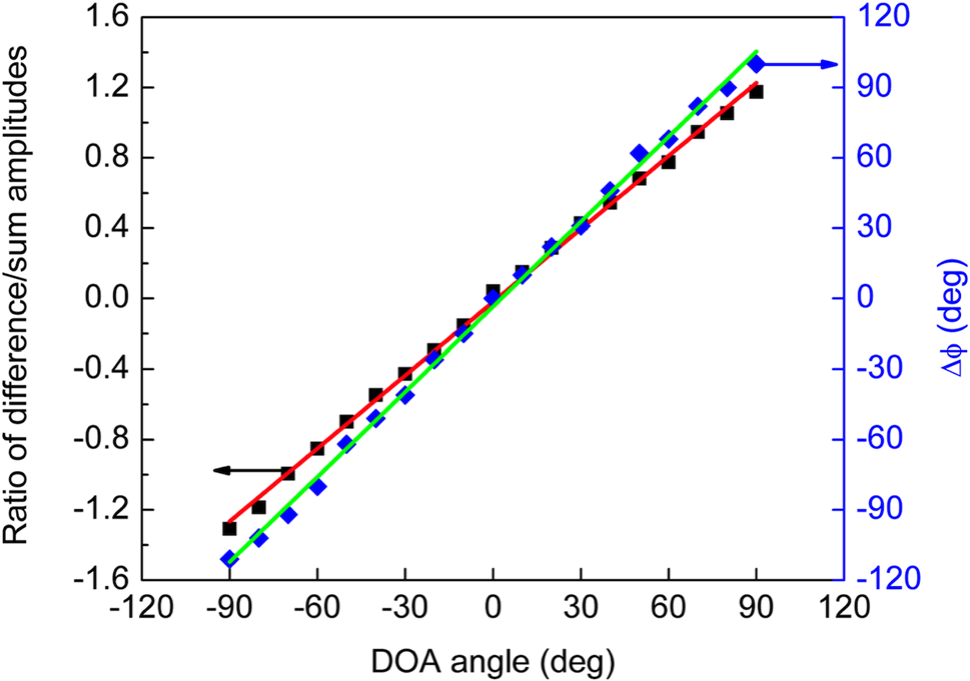
Sensor readouts versus direction of arrival. Measured phase shift between signals from left- and right-wings (blue diamonds) and ratio of difference over sum amplitudes (black squares) as a function of direction of sound arrival. The amplitude ratio is read on the left vertical axis while phase shift on the right. Solid lines, red and green, are the linear fittings for the ratio and phase shift, respectively.

Figure 9a, b show the result of this procedure for their respective counterparts presented in Fig. 8a, b, normal incidence and + 30° incidence. Based on Eq. (5), amplitudes of the sum and difference signals in Fig. 9 are proportional to the bending ($A_{b}$) and rocking ($A_{r}$) mode amplitudes, respectively. Amplitudes of the auxiliary signals for each angular position set on the turntable were determined. Additionally, the ratio of the difference amplitude to the sum amplitude was calculated for each value of incident angle. That value, according to Eq. (4), is related to the sound wave direction of arrival. Figure 10 displays the relationship between phase shift versus sound angle of incidence and ratio of difference over sum amplitudes versus sound angle of incidence, obtained from the recorded waveforms.

A linear fit with slope of 1.21 was obtained for the phase shift versus incident angle and the intercept occurred at − 3.45°, which may be attributed to a systematic deviation from the normal incidence in the initial alignment of the sensor assembly. A similar linear behavior with slope of 1.4 × 10^–2^ was observed for the ratio of difference over sum. These results show that either technique can be used in determining the DOA of an acoustic wave using a single MEMS sensor and with full angular range between − 90° to + 90°. Subsequently, lock-in amplifiers, one for each channel, were used to synchronously detect the phase of each wings’ displacement signal relative to the reference signal. The excitation harmonic signal employed for generating sound was used as the reference for both lock-in amplifiers.

**Figure 11:**
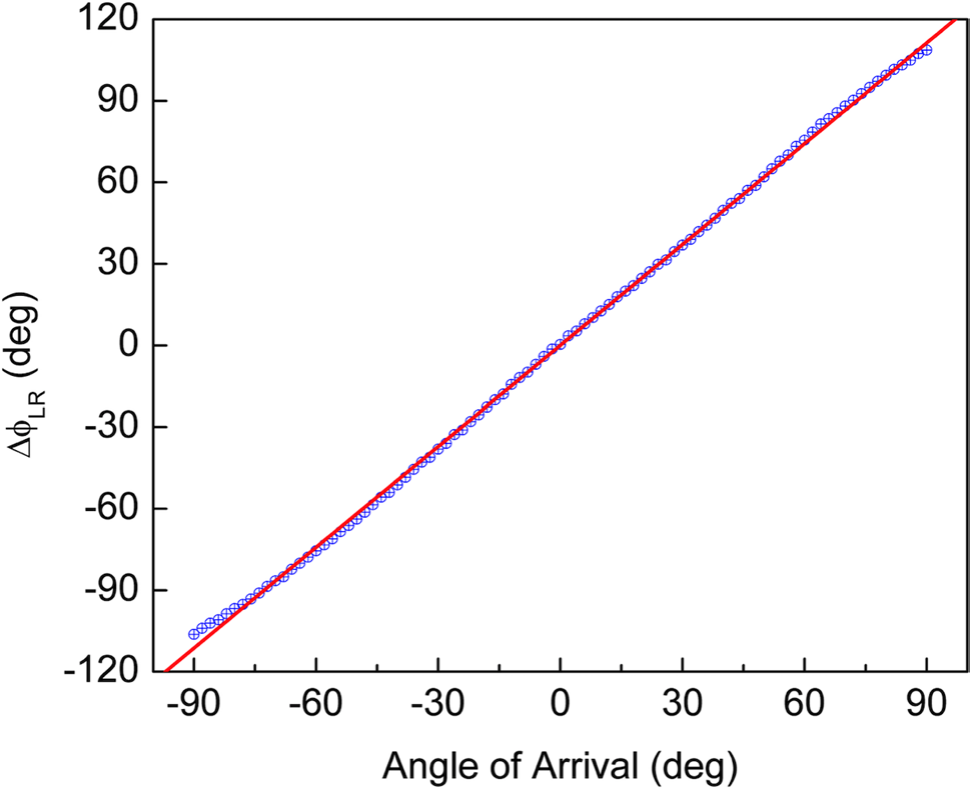
Phase shift between left- and right-wing signals as a function of varying acoustic wave direction of arrival (DOA). Measurements were taken every 2° in the angular range from − 90° to + 90°.

A computer connected to the amplifiers, read the individual phases and calculated their difference. The time constants of the lock-in amplifiers were set to 100 ms and the turntable was rotated from − 90° to + 90° in 2° angular steps. Figure 11 shows the measured phase shift using this approach. A highly linear behavior (R^2^ = 0.99) is obtained, with a slope of 1.24 and shows that synchronous demodulation can be a good option for the sensor readout.

The measurements presented in Figs. 10 and 11 were performed at an acoustic pressure of 500 mPa, which is relatively high. In an effort to determine the detection limits of the phase shift technique, another experiment was performed keeping the distance between the sound source and the sensor constant while electronically adjusting the sound pressure level. The sound pressure level was sequentially reduced and the turntable was rotated in a stepwise manner around the normal incidence position. The phase shifts between the signals associated with the wings displacements were recorded.

**Figure 12:**
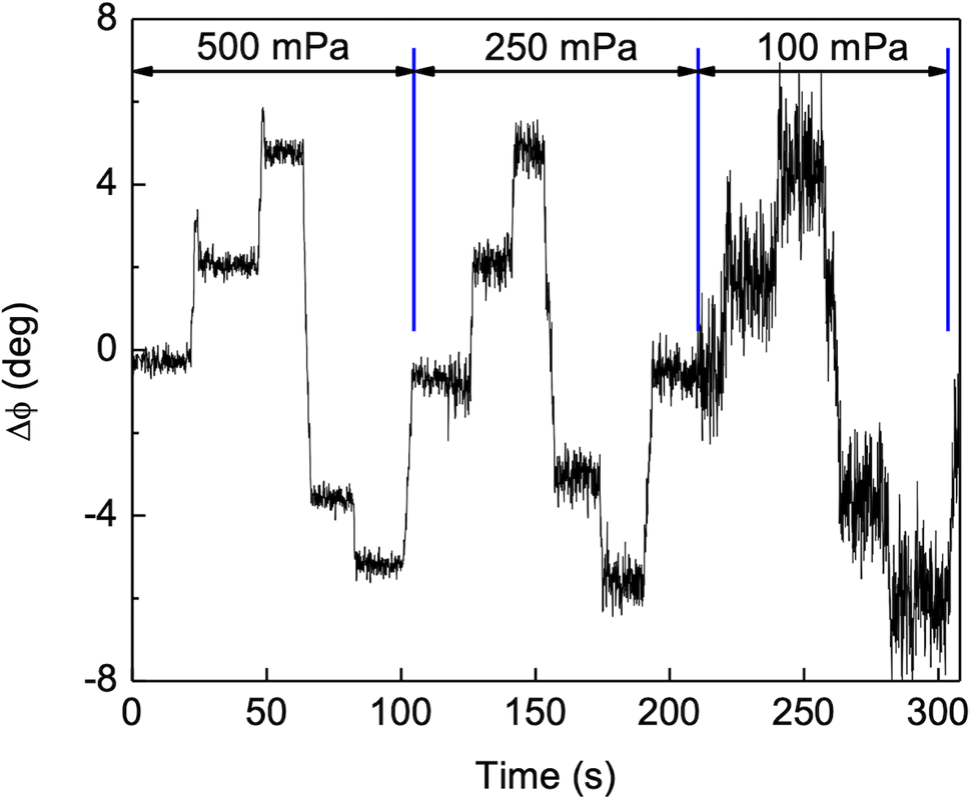
Recorded phase shift time series. Recorded phase shifts between left and right signals for three SPLs (500, 250 and 100 mPa). The time instant on which the SPL was reduced from the previous value is marked by the vertical blue lines. The steps in the recorded signal are due to turntable position changes. Turntable positions are 0°, 2°, 4°, − 2° and − 4°. The increase in the phase shift uncertainty is clearly noticeable after a SPL reduction.

Figure 12 shows the phase shift with time for first three SPL values of 500, 250 and 100 mPa when the angle was stepped in 2° increments. The phase shift uncertainty is determined by the demodulated signal standard deviation (σ), which in turn is related to the angular uncertainty through the linear relationship found between phase shift and AOA. The standard deviation of the angle of arrival (AOA) can be estimated at each SPL using the measured standard deviation for phase shift divided by the slope of the linear fit in Fig. 11. This AOA standard deviation was assumed to be the processing electronics minimum detectable angular shift (angular resolution) for the SPL under consideration.

**Figure 13:**
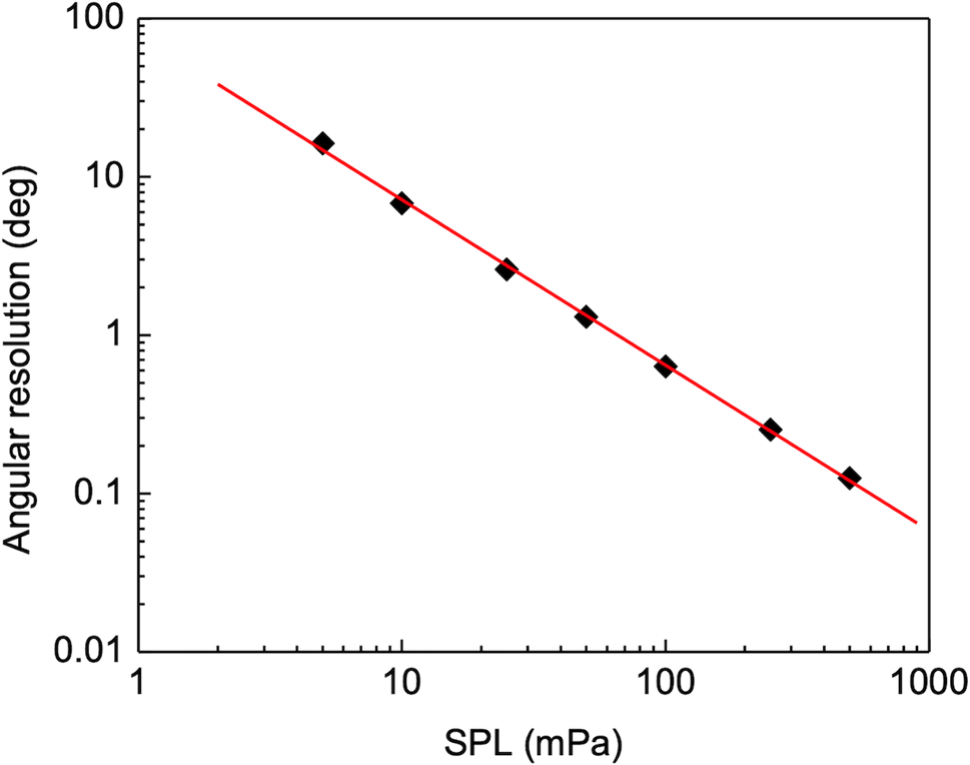
Angular resolution versus SPL. The minimum detectable angle step (angular resolution) is determined from the associated phase shift standard deviation for every SPL applied to the sensor. The linear relationship between phase shift and angle of arrival (Fig. 11) enables the determination of angular orientation uncertainty. A unitary SNR translates into the minimum detectable signal being equal to that uncertainty for each SPL the sensor is subject.

A log–log plot of the equivalent angle error as a function of the incident wave SPL was obtained from these values. The SPL for which the signal to noise ratio would be unitary for a given angular resolution can be determined from it. The minimum detectable angle variation or resolution, obtainable with this detection system for each given value of SPL, can also be determined. Figure 13 shows the calculated angular resolution of the AOA as a function of the sound pressure level applied at the sensor. The linear fit to the data in Fig. 13 gives

11 $$\Delta \theta = 10^{1.9} \left( {SPL} \right)^{ - 1} .$$

As the SPL decreases the angular resolution becomes poorer due to the increase in the phase shift uncertainty. An angular resolution of better than 3° was obtained for SPL greater than 25 mPa (42 dB rel 20 μPa).

## Discussion

In this work, a single biomimetic MEMS directional sensor was used to determine the DOA of an incident sound wave; in contrast to previous works, in which a second measurement by another similar sensor or a calibrated microphone becomes necessary in order to eliminate the sound pressure level (SPL) as one of the problem’s variables. Instead of using two sensors, utilizing the two resonant modes’ responses of the single sensor also eliminates the need for SPL determination, as it was demonstrated theoretically and empirically. Some of the sensor’s geometric parameters had to be conveniently adjusted in order to bring both modal frequency responses closer and enhance their coupling effects, known to be the reason for the directional sensitivity. The rocking mode resonant frequency was measured 1082 Hz and the bending mode, 1510 Hz. Also vital to the single sensor functioning was the reduction of the acoustic pressure communication with the back of the sensor and the cosine dependence of the directivity pattern associated with it. This was accomplished by closing the back of the sensor. The more responsive bending mode was dampened taking advantage of squeezed film effects by putting the sensor on top of a partially closed air cavity. As a result, the bending mode response is spectrally broadened due to a reduction in its Q factor and slightly downshifted, moving closer to the rocking mode response, also favoring their coupling. Laser vibrometry measurements and finite-element method modeling agreed on these trends for a partially closed back cavity and showed a bending mode oscillation sensitivity one order of magnitude less responsive than on the open back case. On the finite-element modeling simulations, the appropriate back air cavity stiffness and damping ratio were adjusted by changing the backplate slit surface area, responsible for the air and acoustic pressure interaction of the cavity with the exterior medium. Mechanical displacements of the wings were transduced to voltage by way of interdigitated air-gap comb finger capacitors placed at the edge of sensor moving wings, a commercially available capacitance-to-digital integrated circuit (PCap04) and an open-source microcontroller unit (Arduino Due) with dedicated embedded software. Simultaneous and independent measurement of the signals associated with each of the wings displacements enabled the experiments validating the use of a single sensor. Spectral response measurements using the electronic signals were found in good agreement with the previously measured and simulated mechanical spectral responses. The obtained overall sensitivity was on the order of 1 V/Pa. Simple signal processing was utilized to determine the DOA from the amplitudes and phases measured simultaneously from both wings, providing two ways of accomplishing it. The phase shift due to a single tone acoustic signal at 1150 Hz presented a linear characteristic with a slope of 1.24 degree/degree [phase shift/angle of incidence]. Further measurements demonstrated that this approach is capable of delivering an angular resolution in the DOA better than 3° for sound pressure levels greater than 25 mPa. It was clear from the results that the distinct character of individual mode interaction with the partially closed back air cavity was crucial for the DOA determination. The mechanical coupling and the pressure difference characteristics due the partially closed back cavity in the biomimetic sensor revealed both to be important. Although not studied in this work, this points in the direction that it should also be the case for the role of the prosternal chamber, the spiracular and tracheal system in the ormia fly hearing organ.

## Methods

### Laser vibrometry

The mechanical spectral responses of the sensor wing displacements with open and closed back configurations were performed using a Polytec OFV-5000 laser vibrometer. The laser head of the OFV 534 unit was installed on an optical breadboard inside the anechoic chamber, while the controller and processing equipment were kept outside, in the chamber’s control room. The sensor holder was also placed on the breadboard right in front of the optical head on a 5-axis motion stage allowing adjustments of the laser beam position on three linear translation directions along with azimuth and elevation angles as well. The position of the beam was visualized with the help of a camera pertaining to the optical head, by means of a splitter collinear with the laser beam. The image was displayed on the control unit screen. The setup also possessed a calibrated omnidirectional microphone model 378A21 from PCB Piezotronics, positioned on the optical breadboard on one side of the device under test, with their longitudinal axes aligned. The signal from the microphone was amplified by a Model 482C sensor signal conditioner, also from PCB Piezotronics. The output of the amplifier was connected to the reference input on the vibrometer junction box and was used by the software to ascertain the sound pressure level impinging on the device under test. The Polytec control software output a sinusoidal signal, frequency swept from 500 Hz up to 2 kHz and amplified by a Techron Power amplifier model 5507 before being connected to a JBL loudspeaker Model No 2450H, 8 ohms impedance, from JBL Incorporated, Northridge, CA, USA, positioned inside the anechoic chamber, making the sound angle of incidence on the sensor close to 45°. Physical impediments in the experiment setup did not allow it to be exactly equal to the desired value. The laser beam was initially positioned at the end of one wing and a measurement obtained by averaging ten frequency swept scans. While keeping all the other positioning and adjustments, the laser beam was positioned at the end of the other wing with the associated ten-average scan measurement obtained.

### Electric spectral response

The closed back sensor was mounted with the circuit board on a post, with the device vertical axis parallel to the post axis, and attached to the mandrel of a Brüel and Kjær controllable turntable type 5960, inside de anechoic chamber while its angular position was remotely adjusted in the control room by a type 5997 turntable controller (Brüel and Kjær). The electronic circuits were powered using a dual output DC power supply model E3620A from Agilent, placed in the control room and connected to the circuit board by coaxial cables threaded through wall feedthrough ducts between both rooms. The excitation swept signal was generated by one of the auxiliary signal outputs of a MLFI Lock-in amplifier, 500 kHz/5 MHz, 60 MSa/s from Zurich Instruments, amplified by a Techron Power amplifier model 5507 before being connected to a fixed JBL loudspeaker Model No 2450H, 8 ohms impedance, from JBL Incorporated, Northridge, CA, USA, positioned inside the anechoic chamber facing the turntable, constituting the sound source for the experiment. The electronic signal coming from one of the sensor channels (wing displacement signal) was connected to the input of this lock-in amplifier, also by threaded cable through the duct, and was synchronously demodulated. Another identical MLFI lock-in amplifier had the signal coming from the other wing connected to its input and demodulating it synchronously and simultaneously with the previous lock-in amplifier demodulation. Both lock-in amplifiers were properly synchronized by connecting their Clock and Trigger inputs/outputs and selecting the one generating the sweep as the master, while the other is configured as a slave. Both instruments were connected to a PC by way of the USB port. The Zurich instruments LabOne software, running on the PC, controls the amplifier parameters and records the data measured. A previous experiment used a calibrated omnidirectional microphone model 378A21 from PCB Piezotronics in place of the directional sensor as a way of relating the SPL incident on it to the lock-in amplifier auxiliary output voltage amplitude. The calibration factor (Pa/V) was utilized to determine the actual SPL once a voltage amplitude was set on for the subsequent measurements. The sensor was positioned at two angular positions with respect to the loudspeaker (± 45°) and for each position at a time the sinusoidal swept signal from 500 Hz to 2 kHz was applied. The calibrated SPL applied was 500 mPa. The magnitude of the signals demodulated by the lock-in amplifiers for the left and right wings were individually recorded and stored in the PC to be plotted later.

### Directional characteristic

Two experiments of this kind were performed. The experimental setup inside of the anechoic chamber was exactly the same as the one described for the electric spectral response above for both. The difference lied on where the signals from the wings (right and left) were connected in the control room. On the first experiment each of the signals were connected to a channel of a DSO7054B InfiniiVision digital storage oscilloscope from Agilent Technologies. A single tone acoustic signal with frequency equal to 1150 Hz was applied to the loudspeaker with amplitude such that it was produced an SPL of 500 mPa, according to the previously performed calibration. The sensor angular position was adjusted by the turntable to cover the interval [− 90°, 90°] angles of incidence in 10° steps. Waveforms were saved to a flash drive connected to the oscilloscope USB port to be post processed. The phase shift between the signals was determined at the zero-crossing for each of the signals. On the second experiment, the signals were each connected back to the same lock-in amplifiers they were before. The experiment was then repeated but now with the angular steps reduced to 2° over the same interval. The lock-in amplifiers detected the individual phase of each signal (left or right wing) with respect to the single tone excitation used as a common synchronized reference signal that was generated internally in the master lock-in amplifier. Individual phases were subtracted in the instrument controlling PC with the LabOne software to produce the phase shift between the two demodulated signals.

### Detection limits

In order to determine the detection limits of the phase shift approach, the same setup for the second experiment of directional characteristic was utilized. Instead of keeping the incident sound pressure level constant, it was electronically and sequentially adjusted to 500, 250, 100, 50, 25, 10 and 5 mPa. At each SPL value, the turntable was rotated in a stepwise manner at five rotation positions placed symmetrically on and around the normal incidence position (0°), being held at each angular position for about 20 s. The initial angular step was set to 2°. The phase shifts were determined by the same method described before and the time series for the values was stored in the computer. For each step, the average value of phase shift (μ) and its standard deviation (σ) were calculated. Assuming that the minimum angular step on the turntable that would allow the electronic system to discriminate the two signal levels is equal to one standard deviation on the detected signal, or a unitary signal to noise ratio (SNR), each SPL will have an associated minimum detectable signal, being that which the signal standard deviation equal to the difference between averages of two adjacent angular steps. If the minimum detectable signal was reached for a given SPL, meaning that it was not possible to discriminate the angular step in the detected signal time series after the turntable had been rotated by one current angular step, then the angular step was increased and kept for the subsequent (lower) SPL, until it would not be possible to discriminate from the noise and the process repeated. The angular step had to be increased to 5° for the 25 mPa SPL and to 10° for the 5 mPa SPL. This way, the phase shift uncertainty related to an SPL is determined. But the uncertainty sought is the one in the angle of arrival, which is related to the phase shift uncertainty through the line slope determined in the second directional characteristic experiment (1.237). Dividing the phase uncertainty by the line slope results the angle of arrival uncertainty related to each SPL.

## Supplementary information

Supplementary Figures

## Data Availability

The datasets generated during and/or analyzed during the current study are available from the corresponding author on a reasonable request.
