# Supplementary material for: Electronic phase shift measurement for the determination of acoustic wave DOA using single MEMS biomimetic sensor
Source: Sci Rep. 2020 Jul 29;10:12714. doi: 10.1038/s41598-020-69563-1 (PMC7391735; doi:10.1038/s41598-020-69563-1)
Supplement: Supplementary file 1 — Supplementary Figures [file 41598_2020_69563_MOESM1_ESM.docx]

Electronic phase shift measurement for the determination of acoustic wave DOA using single MEMS biomimetic sensor

Renato C. Rabelo^*^, Fabio D. Alves and Gamani Karunasiri

*Department of Physics, Naval Postgraduate School, Monterey, CA 93943, USA.*

*rcrabelo@nps.edu*

**Supplementary information**

| 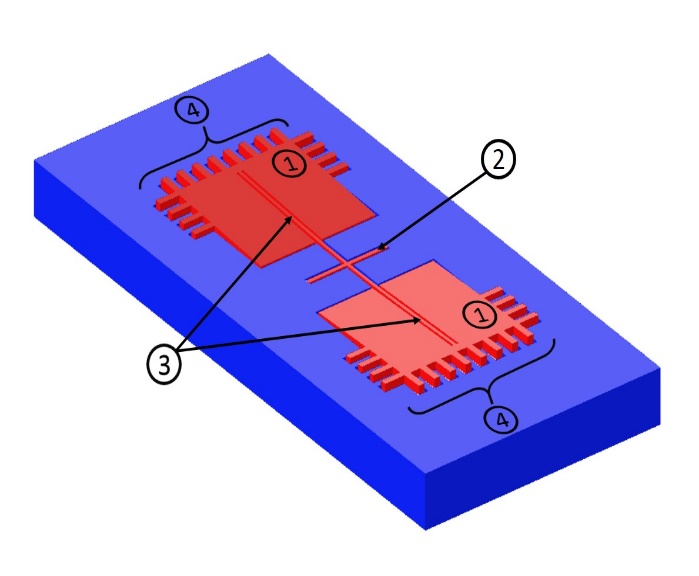 | 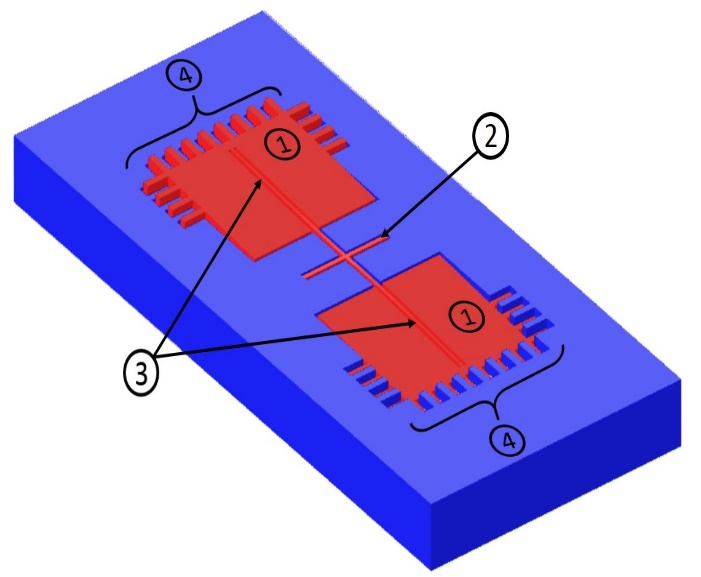 |
| --- | --- |
| (a) | (b) |

**Figure S1** – 3D Schematic diagrams of the device. Wing displacement schematics associated with the (a) bending mode; (b) rocking mode. The displacements are not proportional to device size and were exaggerated to become perceivable in the diagrams. Structural features were numbered using the same sequence presented in Fig.1 of the main text:
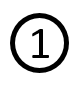
 Wings;
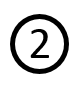
 Leg;
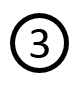
 Bridge;
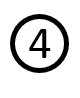
 Interdigitated comb finger capacitors. It is noticeable that the wings are displaced in the same sense for the bending mode (in-phase) and in an opposite sense (out-of-phase) for the rocking mode. On both diagrams, the blue block represents the sensor die and the red surface, the freestanding membrane. The only extension of the membrane not trenched out and anchoring it to the substrate are the smaller sides or the rectangular red surface associated with the leg (
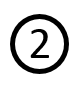
).


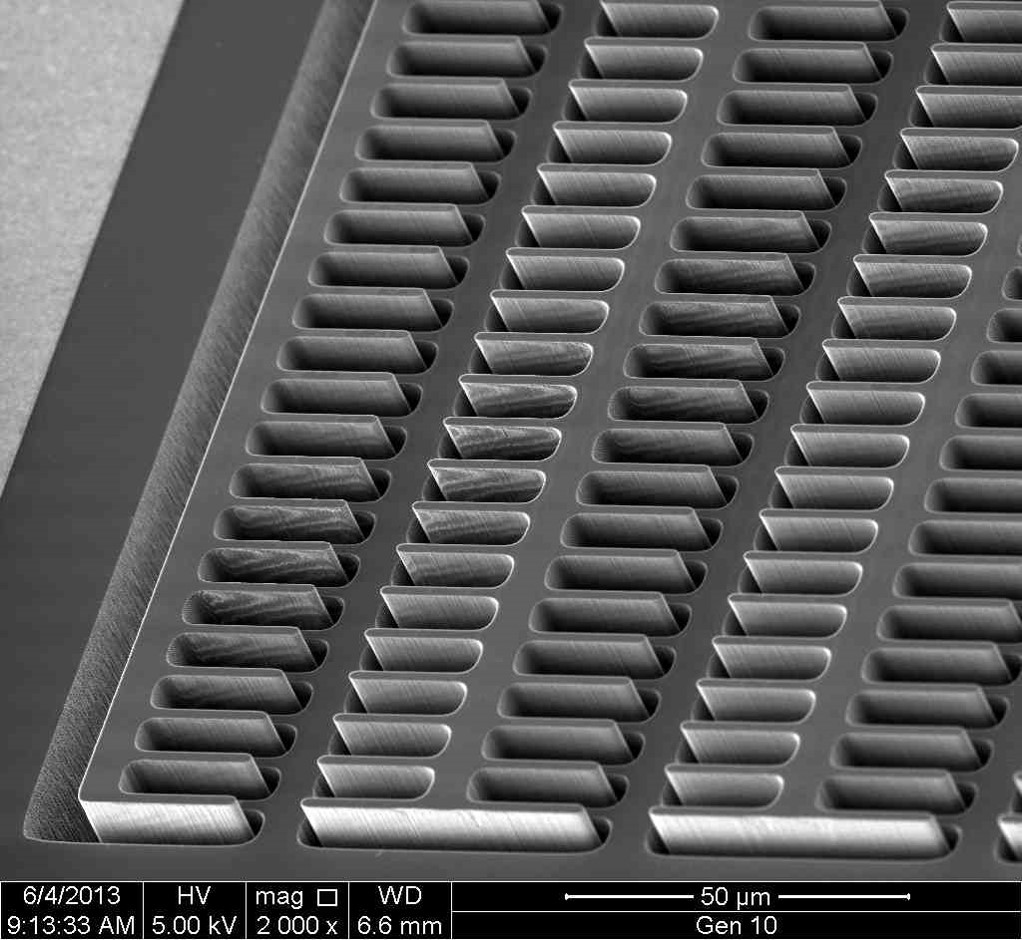


**Figure S2** – Device SEM image. Detail of the interdigitated comb fingers at the corner edge of a wing.
